# Bromide of Ethyl as an Anæsthetic in Labor

**Published:** 1885-07-20

**Authors:** 


### BROMIDE OF ETHYL AS AN AN ANÆSTHETIC IN LABOR.

Dr. Montgomery, reviewing the various anæsthetics, said chloroform is objectionable in that it causes inertia uteri and tedious labor and increases the danger of post partum hemorrhage. The relatively infrequent fatal cases under its surgical practice, and the still more rare serious results from its use in obstetrics, forbid its habitual use. The use of ether in natural labor is infrequent, because to relieve pain the patient must be profoundly etherized. Partial etherization but destroys the ability to bear pain without obtunding sensation. Besides, Tait has demonstrated that ether passes rapidly into the circulation of the fetus, endangering its existence. The mixture of nitrous oxide and air, advocated by Tclikowitsch, requires a special apparatus and is unweildy. The ideal anæsthetic is one that is safe for mother and child, certain in effects, rapid in relieving pain without loss of consciousness, and whose effects pass off quickly. All these demands are met by the bromide of ethyl. He enumerated 112 cases in which it had been used, 29 of which were in his own practice: none of the mothers died, and but three of the children. In none of the latter could death be attributed to its use. It was administered during the second stage of labor by laying a napkin wet with the ethyl over the face of the patient at the advent of each pain, and withdrawing it as the pain subsides. Unless a drachm was used, the sensation of pain was obtunded without arresting consciousness. The process of labor was carried forward vigorously and quietly, the patient ready to exert or withold voluntarily aid as the attendant might direct, and the expulsion of the head was attended with no greater pain than accompanies the evacuation of obstinately constipated bowels. His experience did not lead him to believe that its use would induce uterine inertia or increase the tendency to post partum hemorrhage.

Dr. D. M. Barr is much interested in this subject, and thinks from this report that the indications for the usefulness of bromide of ethyl are favorable. He would like to know to what degree the patient returned to consciousness between the pains. He will use bromide of ethyl as an experiment, but he will say here that his old combination still gives him the very best effects with perfect safety. He has continued to use it in almost every case of labor since his report on anæsthetics in labor, (see Med. and Surg. Reporter, March 13, 1880.) He would feel some hesitation in using bromide of ethyl, as it is dangerous, even if not so much so as chloroform. How would it act if mixed with ether? The objectionable qualities of ether and chloroform balance and overcome each other, and the addition of alcohol to the mixture prevents their explosive action, so to say, that is, the sudden effect of a full and deep inspiration of the strong vapor of the anæsthetics is prevented by the admixture of chloroform in proportion of chloroform one part by measure, ether three parts, and alcohol two parts. If a portion of this mixture be placed in a saucer and heated, it will all evaporate together; the ether does not evaporate from the alcohol. This mixture soothes the pain and makes the patient happy. The excited or drunken stage of ether is avoided, the danger of chloroform is avoided; unconciousness is unnecessary, and is not produced. If bromide of ethyl has any of the dangers of chloroform, they are unchecked by admixture with correcting agents. Dr. Barr related the particulars of a case in which he had employed his mixture, to show how kindly it acted in presence of suspected functional heart disease. In this case its administration was continued for nine hours; the pain was relieved, there was no vomiting, no effect upon the pulse.

Dr. R. P. Harris remarked that chloroform was considered perfectly safe as an anæsthetic in labor. Playfair in his last book advocates it on this ground, but there have been fatal cases. The danger of an anæsthetic can only be ascertained after it has been used and reported by many observers, so as to get an average. Some men are careful and cautious, others are not. The use of mixed anæsthetics is becoming more general in England.

Dr. Wm. M. Welsh, upon invitation by the President, remarked that he had no experience with bromide of ethyl, and but very little with other anæsthetics. He had given ether in one case, and a troublesome condition of intoxication had been developed. He was applying the forceps, when the woman suddenly seized one blade and wrenching it out of the vagina tore the vulva. He prefers to get along without the use of anæsthetics.

Dr. Henry Leaneau, upon invitation by the President, said he also had no experience with bromide of ethyl. He does not hesitate to use ether in bad cases of labor, but he does not employ it if he can avoid it. He is not yet convinced that the use of anæsthetics in natural labor is advisable. There has not yet been formulated a satisfactory definition of natural labor. He is now engaged in studying that subject. He has observed in private practice most of the positions described by Engelmann in his article on primitive obstetric practice. The sympathetic system in labor reacts upon the cerebro-spinal system and produces the condition of nervous excitement, which is seen in the patient, and which often extends to the friends of the patient, and even to the doctor in charge of the case. After a close observation of over six hundred cases, he thinks labor can be as natural a physiological action as respiration or circulation of the blood, or any other function of the body, with this one difference, that whereas the ordinary performance of function is pleasurable, in labor pleasure is replaced by pain. He is not in favor of the use of anæsthetics merely to obtund or remove this pain, as it is natural, and anæsthetics may interfere with the natural process, and cause relaxation or retard involution, resulting in that root of endless misery, a subinvoluted uterus.

He has been making careful studies with a dynamometer, which measures the available pressure. I believe that it gives the sum of the pressure applied to the ovum expressed in the projection of the advancing part, against which it rests. He will continue these studies, which are not yet complete. The force of the accessory muscles, as the diaphragm and abdominal muscles, takes no real part in the expulsion of the fetus, but merely embraces the uterus, preventing any rebound or loss of force when the ovum impinges against the pelvic walls or perineum. Their action being to sustain or hold the uterus closely to its work. The entire force exerted is not nearly so great as is usually supposed. The force of labor does not exceed that of arterial pressure, which I think is about six pounds. The exit of the child is not violent but gradual. It is a popular belief that a majority of labors occur at night, but, of his six hundred carefully recorded cases an equal number were born between 6 a. m. and 6 p. m., and between 6 p. m. and 7 a. m. There are, however, two acmes, one at 11 p. m. and another between 7 and 8 a. m. These two periods correspond to the times of greatest blood pressure. He ranges himself on the side of those who believe in the non-necessity of the use of anæsthetics in natural labor.

Dr. W. H. Parrish would like to hear more particularly from Dr. Montgomery in his closing remarks respecting the safety of bromide of ethyl as an anæsthetic. A few years ago it was introduced into surgical practice in this city, and was abandoned in consequence of its dangerous character. If it is dangerous in surgery why should it not be so also in obsteterics? Chloroform, which was at one time considered perfectly harmless in the latter class has been found to be no safer there than in ordinary cases.

He has established for himself three rules respecting the use of anæsthetics in obstetric cases:

1st. In any normal case no anæsthetic is required.

2d. If the patient is nervous, excited and uncontrollable, he gives chloroform at the incipiency of each pain, to quiet the excitability of the patient and take off the sharpness of the pain without producing unconsciousness; during the intervals between pains the chloroform is withheld.

3d. Whenever he considers that unconsciousness, full anæsthesia, is necessary he employs ether so as to avoid the depressing effects of chloroform. Bromide of ethyl might be used in place of chloroform, as indicated in his second rule, if shown to be equally safe; but he would not consider it proper to use it to produce complete relaxation as required for version or the application of the forceps. Prof. Wood, in his experiments, found bromide of ethyl more dangerous than chloroform. Dr. Parrish does not now use it, and he fears it would go hard with any physician before our courts if he had a fatal accident occur during its use.

Dr. Baer thought Dr. Leaman's estimate of the force required to extrude a full term fetus far below the mark. A child weighing seven pounds or over is pushed through a curved, horizontal, resisting passage, with irrisistable power; and sometimes rapidity. A force of six pounds would not lacerate a perineum. In some cases there is but slight resistance, and the force required is small and the pain not severe. He has seen cows moaning from the severity of their pains during parturition. In one case recently a cat was delivered of its first kitten after severe and prolonged pain and the second kitten required three hours for its extrusion. The pains of labor are more easily alleviated than the pains of surgery by anæsthetics and with no increase if not a diminution of danger.

Dr. Montgomery, in closing, said that as to danger from the use of bromide of ethyl, he thought there was no danger if a pure article was used. The patient is not completely narcotized, consciousness is not lost; the administration of the drug is interrupted. The patient can co-operate although relieved of suffering. She can answer questions. Prof. Muller is the only one who has failed in obtaining good effects, and this was probably due to impurity in the drug. Bromide of ethyl does not take place of chloroform, nor does it produce muscular relaxation, nor relaxation of the uterus as required in version. It can be pushed to complete unconsciousness, but that is not necessary as pain will be relieved without while the contractions of the uterus and respiratory muscles are fully as effective as without it. Labor is undoubtedly a physiological process as much so as respiration or defecation, but it does hurt. It is the type of the most severe and agonizing suffering, and we as physicians are called on to relieve that suffering and prevent the waste of vital force to the extent that we can by preventing pain, long continued pain. Bromide of ethyl is apparently entirely safe when given as I have used it. Experimental physiologists do not all agree with Prof. Wood as to the comparative danger of this and other anæsthetics.—*Obstet. Soc. Phil.*, *Ob. Gazette.*

